# Association of the leptin receptor Q223R (rs1137101) polymorphism with obesity measures in Sri Lankans

**DOI:** 10.1186/s13104-020-4898-4

**Published:** 2020-01-16

**Authors:** Y. A. Illangasekera, P. V. R. Kumarasiri, D. J. Fernando, C. F. Dalton

**Affiliations:** 1 Dept. of Pharmacology, Faculty of Medicine, University of Peradeniya, Peradeniya, Sri Lanka; 2 Dept. of Community Medicine, Faculty of Medicine, University of Peradeniya, Peradeniya, Sri Lanka; 3 Dept. of Diabetes and Endocrinology, Kings Mill Hospital, Sherwood Forest Hospitals NHS Foundation Trust, Sutton-in-Ashfield, UK; 4 Biomolecular Sciences Research Centre, Faculty of Health and Wellbeing, Sheffield Hallam University, Sheffield, UK

**Keywords:** Obesity, Genetics, Polymorphism, Leptin receptor

## Abstract

**Objective:**

The role of genetic factors in the development of obesity is largely unreported in Sri Lankans. The Q223R (rs1137101) single nucleotide polymorphism (SNP) of the leptin receptor (*LEPR*) gene has been associated with obesity measures in various ethnicities. We investigated the association of the Q223R polymorphism with obesity related anthropometric measures and biochemical parameters fasting blood glucose and lipid profile in a sample of 530 Sri Lankan adult subjects (age 18–70 years) representing both urban and rural areas of residence.

**Results:**

The *LEPR* Q223R variant G allele frequency was 0.54. The polymorphism was associated with body mass index (*p *= 0.04) and waist circumference (*p *= 0.02) measures in overweight and obese (BMI ≥ 25 kgm^−2^) subjects with the variant allele conferring a greater risk of adiposity. Residency in urban areas eliminated the protective effect of the non-risk genotype (AA) in the development of obesity.

## Introduction

Obesity is currently a global epidemic and Sri Lanka too has seen a dramatic rise in the rates of obesity over the past 3 decades [1]. Rapid advances in the fields of genetics and molecular biology have lead to the discovery of many genetic variants associated with obesity and adiposity related quantitative traits. However the effect of common genetic variants in the development of complex obesity is largely unreported in Sri Lankans.

The leptin hormone is secreted in proportion to the adipose tissue mass and indicates the nutritional status to the brain. Leptin acts through the leptin receptors which are located in the hypothalamus which is the central regulatory centre of energy homeostasis and appetite regulation. The leptin receptors are encoded by the *LEPR* gene located in chr1p31. Mutations of the *LEPR* gene are associated with monogenic forms of severe early onset obesity and hyperphagia [2]. A number of SNPs of the coding region of the *LEPR* gene have been described [3]. Of these the Q223R (dbSNP:rs1137101) polymorphism occurs as a result of a non-conservative A to G substitution at codon 223 resulting in a glutamine to arginine amino acid change. This functional variant reduces leptin binding and thus impairs leptin signaling [4]. Numerous studies in various populations have replicated the association with Q223R SNPs with obesity measures with the variant G allele conferring a greater risk [5, 6]. Through the present study we aimed to replicate the association between the *LEPR* Q223R SNP with obesity related anthropometric measures and biochemical measures in a Sri Lankan population.

## Main text

### Methods

#### Subjects

The study was conducted in the Kandy district of Sri Lanka. A multi-stage random sampling method using names from electoral lists was used to select 530 study subjects (age 18–70 years) from the adult general population representative of 6 administrative divisions covering both urban and rural areas of residence. Pregnant women, women up to 3 months postpartum and those suffering from pathological forms of obesity were excluded from the study by examining medical records where available.

#### Genotyping

DNA was extracted from whole blood using QIAamp DNA Midi kits (QIAGEN, Germany) according to the manufacturer’s protocol. Genotyping was performed by real-time PCR and allelic discrimination using Taqman assays (Applied Biosystems, Foster city, CA). Real-Time PCR was performed in a 96-well format in a total 10 µl reaction volume using VIC/FAM dye labelled allelic probes with Taqman GTXpress Master Mix. The reaction mixture was subjected to a standard thermal protocol of 95 °C for 10 min, 40 cycles of 95 °C for 15 s and 60 °C for 1 min in the StepOnePlus© Thermocycler (Applied Biosystems, Foster city, CA). Genotyping quality control was performed by duplicating each sample and including two negative controls for each 96-well plate.

Serum biochemistry analysis for FBS (Fasting Blood Sugar) and lipid profile parameters was conducted using the Indiko™ (Thermo Fisher Scientific, USA) fully automated clinical and specialty chemistry analyser. Biochemical analysis was performed within a few hours of samples collection.

#### Data analysis

Statistical analysis was performed using SPSS ver.17 (IBM) software. Genotype numbers were determined manually from the allelic discrimination plots and allele frequencies were determined from the genotype frequencies. The genetic association analysis was performed assuming an additive genetic model. Calculation for deviation from Hardy–Weinberg equilibrium was performed using Pearson’s Chi square test. Non-normal distributions of data were corrected by log transformation. Comparisons of means were performed using the student’s t-test, ANOVA or ANCOVA models where covariate adjustments were required. We also investigated the potential moderating effect of area of residence (urban/rural) on the association between SNPs and body mass index (BMI). Thus two-way factorial ANOVA was used to investigate interactions. Overweight and obesity were defined as a BMI ≥ 25 kgm^−2^ and BMI ≥ 30 kgm^−2^ respectively. All *p* values < 0.05 were considered significant.

## Results

The study sample comprised of 324 (61.1%) females. Overweight or obesity was observed in 264 (49.8%) of the study population. The baseline characteristics of the study population are displayed in Table 1. Significant gender differences in waist-to-hip (WHR), high density lipoprotein (HDL) and triglyceride (TGL) values were observed with higher values in males for WHR and TGL. Rural populations represented 71.6% of the study cohort. Urban (U) dwellers recorded significantly higher obesity measures compared to rural (R) dwellers for BMI (U:25.7 ± 4.3 kgm^−2^, R:24.7 ± 4.1 kgm^−2^, *p *< 0.05), Waist Circumference (WC) (U:92.2 ± 10.7 cm, R:88.1 ± 10.6 cm, *p *< 0.01) and WHR (U:0.94 ± 0.07, R:0.91 ± 0.07, *p *< 0.01).

**Table 1:** Baseline characteristics of study population

	Male (n = 206)	Female (n = 324)	*p*	Total (530)
Age (years)	47.6 ± 11.0	47.5 ± 11.3	0.86	47.5 ± 11.2
BMI (kg/m^2^)	24.7 ± 3.8	25.2 ± 4.4	0.13	25.0 ± 4.2
WC(cm)	90.0 ± 10.4	88.7 ± 11.0	0.18	89.2 ± 10.8
WHR	0.95 ± 0.06	0.91 ± 0.07	*< 0.001*	0.92 ± 0.07
FBS (mmol/l)	5.4 ± 2.5	5.5 ± 3.7	0.77	5.5 ± 3.2
TC (mmol/l)	5.1 ± 1.1	5.8 ± 1.6	0.30	5.7 ± 1.5
LDL-C (mmol/l)	3.8 ± 2.4	3.8 ± 2.1	0.88	3.8 ± 2.2
HDL-C (mmol/l)	1.4 ± 1.7	1.4 ± 0.3	*< 0.001*	1.4 ± 1.1
TGL (mmol/l)	3.5 ± 1.7	3.2 ± 2.1	*< 0.05*	3.4 ± 2.0

Data shown as mean ± SD; FBS, HDL-C and TGL log transformed for analysis

*BMI* body mass index, *WC* waist circumference, *WHR* waist-to-hip ratio, *FBS* fasting blood sugar, *TC* total cholesterol, *LDL-C* low density lipoprotein cholesterol, *HDL-C* high density lipoprotein cholesterol, *TGL* triglycerides

The variant G allele frequency was 0.54. The observed genotype frequencies did not show significant deviation from the Hardy–Weinberg equilibrium (*p *= 0.44). There were also no significant differences in the distribution of genotype frequencies between genders (*X*^2^ (2) = 1.32, *p* = 0.52).The association between *LEPR* Q223R and obesity and biochemical measures in the overall and overweight or obese population are displayed in Table 2. In the overall study population as well as in gender specific subgroup analysis, no significant associations between *LEPR* genotypes and BMI, WC, WHR or biochemical parameters were found. However when the analysis was conducted in overweight and obese subjects only (BMI ≥ 25 kgm^−2^), significant differences in BMI (*p *= 0.04) and WC (*p *= 0.02) values were observed. Post hoc analysis with Bonferroni correction revealed that homozygous carriers of G allele (Arg/Arg) recorded significantly greater BMI values (M(SE) = 1.3 kgm^−2^ (0.53)) compared to the AA (Gln/Gln) carriers (*p *= 0.04). The GG carries recorded significantly higher WC values compared to the AG carriers (M(SE) = 2.8 cm (1.1), *p *= 0.04) as well as the AA carriers (M(SE) = 3.8 cm (1.4), *p *= 0.03). No significant associations were observed when the analysis was conducted for the obese group based on the World Health Organization (WHO) definition of BMI ≥ 30 kgm^−2^.

**Table 2:** Association of *LEPR* rs1137101 with obesity measures and metabolic parameters in the overall study group and overweight or obese subjects (BMI ≥ 25 kgm^−2^)

	Overall study group					Overweight or obese (BMI ≥ 25 kgm^−2^)				
	AA n = 107	AG n = 272	GG n = 151	*p*	p*	AA n = 53	AG n = 141	GG n = 70	*p*	p*
BMI (kgm^−2^)	24.4 ± 4.1	25.1 ± 4.1	25.2 ± 4.5	0.28	0.31	27.6 ± 2.6	28.2 ± 2.8	29.0 ± 3.3	*0.03*	*0.04*
WC(cm)	88.0 ± 10.7	89.4 ± 10.6	89.8 ± 11.3	0.41	0.36	95.2 ± 7.4	95.9 ± 7.7	98.4 ± 8.4	*0.04*	*0.02*
WHR	0.92 ± 0.07	0.92 ± 0.07	0.92 ± 0.06	0.78	0.94	0.94 ± 0.07	0.94 ± 0.07	0.94 ± 0.06	0.89	0.94
FBS (mmol/l)	5.3 ± 2.5	5.6 ± 3.9	5.2 ± 2.0	0.45	0.65	5.2 ± 1.9	5.6 ± 2.5	5.4 ± 2.2	0.53	0.52
TC (mmol/l)	5.5 ± 0.9	5.8 ± 1.2	5.8 ± 2.1	0.41	0.45	5.4 ± 1.0	5.8 ± 1.3	5.9 ± 2.7	0.18	0.19
LDL-C (mmol/l)	3.5 ± 0.9	3.8 ± 1.7	4.0 ± 3.3	0.19	0.19	3.4 ± 1.0	3.8 ± 2.2	4.5 ± 4.7	0.08	0.09
HDL-C (mmol/l)	1.3 ± 0.3	1.5 ± 1.5	1.4 ± 0.3	0.35	0.52	1.3 ± 0.3	1.4 ± 0.3	1.4 ± 0.3	0.16	0.38
TGL (mmol/l)	3.3 ± 1.6	3.4 ± 1.7	3.4 ± 2.6	0.49	0.56	3.3 ± 1.4	3.6 ± 1.8	3.8 ± 3.3	0.62	0.45

Data shown as mean ± SD; FBS, HDL-C and TGL log transformed for analysis

*BMI* body mass index, *WC* waist circumference, *WHR* waist-to-hip ratio, *FBS* fasting blood sugar, *TC* total cholesterol, *LDL-C* low density lipoprotein cholesterol, *HDL-C* high density lipoprotein cholesterol, *TGL* triglycerides

*p* unadjusted; p* adjusted for age and gender

The effect of area of residence on the relationship between *LEPR* status and BMI in the total study population was investigated. The variant allele carriers were considered as a single group (GG + AG) and compared against the non-risk (AA) group for the analysis. Whilst the risk allele carriers (GG + AG) had significantly greater BMI values compared to the AA carriers in rural populations (mean difference = 1.2 kgm^−2^ (95%CI 0.15–2.3), *p *= 0.03) this difference was not observed in the urban group. Whilst both genotype groups showed greater BMI values in urban populations, the non-risk (AA) genotypes showed a highly significant increase BMI value in urban areas (Mean difference = 2.2 kgm^−2^ (95%CI 0.5–3.9), *p *< 0.01) whilst the risk genotypes (GG + AG) showed an increase which was marginally insignificant (BMI difference = 0.8 kgm^−2^ (95%CI − 0.1–1.7), *p *= 0.08).

### Discussion

We conducted a population based study to investigate the potential association between the *LEPR* Q223R SNP and obesity related measures in Sri Lankans. The study sample was recruited randomly from the adult general population and represented a wide age range of 18–70 years. The sample also represented residents from both urban and rural populations. These facts make the findings of the study highly generalizable to the true population. Our results demonstrate that the presence of the variant `G allele’ of the *LEPR* Q223R polymorphism is associated with greater BMI and WC measures and these associations are most prominent in subjects with a BMI greater than 25 kgm^−2^. Similar results have been reported previously where the association between the `G’ allele and obesity related anthropometric measures were observed only in obese subjects [7]. When the entire study population was analyzed a trend for increasing BMI and WC was evident with the `G’ allele carriers though these associations did not reach statistical significance. Our findings indicate that the effects of the *LEPR* polymorphism on obesity is accentuated in higher BMI groups and that factors which are potentially protective against obesity (i.e. physical activity, diet) may attenuate the obesogenic effects of the gene variant. Though the sample size was somewhat moderate in the present study positive associations between the *LEPR* Q223R polymorphism and obesity have been reported even in smaller study groups in other ethnic populations [7, 8]. Therefore the results indicate a degree of ethnic heterogeneity in the effect of the polymorphism.

In the analysis of association with biochemical parameters we found no association between the *LEPR* polymorphism and FBS or lipid measures. The *LEPR* Q223R polymorphism has been reported to be associated with FBS and lipid measures in certain studies [7, 9, 10]. It should taken into perspective that due to the random selection of the study group, individuals currently on pharmacotherapy for type 2 diabetes and dyslipidaemia were also represented. In addition the biochemical measures tested are also influenced by lifestyle factors such as diet and physical activity levels. Therefore potential association with the polymorphism may have been masked to a certain extent due to the aforementioned factors.

Finally we also investigated whether the effect of *LEPR* Q233R SNP genotype on obesity was influenced by urban/rural living. Our results demonstrated that benefits of having a lower BMI with the non-risk genotypes (AA) was only present in rural populations and that urban living significantly narrowed the difference in BMI between the risk vs. non-risk genotypes. Therefore it could be postulated that factors unique to urban living may negate the beneficial effects of carrying the non-risk *LEPR* genotype. In fact urban living is known to be an independent risk factor for obesity in Sri Lankans [11]. The investigation of the mediating effects of factors such as diet and exercise on the association between *LEPR* polymorphisms and obesity outcomes is warranted in future studies.

In conclusion our study demonstrates that the *LEPR* Q223R SNP is associated with obesity measures in Sri Lankan populations and that the effects of the genotype vary according to the urban and rural areas of residence.

## Limitations

The moderate sample size may have contributed to the lack of association seen in the overall study group. The effects of genetic variants on complex obesity are often small and require large sample numbers to detect statistically significant effects.

## Data Availability

The data sets of the current study are available from the corresponding author on reasonable request.

## Abbreviations

SNP: single nucleotide polymorphism
LEPR: leptin receptor
BMI: body mass index
WC: waist circumference
WHR: waist to hip ratio
FBS: fasting blood sugar
TC: total cholesterol
LDL-C: low density lipoprotein cholesterol
HDL-C: high density lipoprotein cholesterol
TGL: triglycerides
